# Sleep Tracking of a Commercially Available Smart Ring and Smartwatch Against Medical-Grade Actigraphy in Everyday Settings: Instrument Validation Study

**DOI:** 10.2196/20465

**Published:** 2020-11-02

**Authors:** Milad Asgari Mehrabadi, Iman Azimi, Fatemeh Sarhaddi, Anna Axelin, Hannakaisa Niela-Vilén, Saana Myllyntausta, Sari Stenholm, Nikil Dutt, Pasi Liljeberg, Amir M Rahmani

**Affiliations:** 1 Department of Electrical Engineering and Computer Science University of California Irvine Irvine, CA United States; 2 Department of Computing University of Turku Turku Finland; 3 Department of Nursing Science University of Turku Turku Finland; 4 Department of Obstetrics and Gynaecology Turku University Hospital Turku Finland; 5 Department of Public Health University of Turku and Turku University Hospital Turku Finland; 6 School of Educational Sciences and Psychology University of Eastern Finland Joensuu, Kuopio Finland; 7 Centre for Population Health Research University of Turku and Turku University Hospital Turku Finland; 8 Department of Computer Science University of California Irvine Irvine, CA United States; 9 School of Nursing University of California Irvine Irvine, CA United States

**Keywords:** sleep, smart ring, smartwatch, actigraphy, wearable technology

## Abstract

**Background:**

Assessment of sleep quality is essential to address poor sleep quality and understand changes. Owing to the advances in the Internet of Things and wearable technologies, sleep monitoring under free-living conditions has become feasible and practicable. Smart rings and smartwatches can be employed to perform mid- or long-term home-based sleep monitoring. However, the validity of such wearables should be investigated in terms of sleep parameters. Sleep validation studies are mostly limited to short-term laboratory tests; there is a need for a study to assess the sleep attributes of wearables in everyday settings, where users engage in their daily routines.

**Objective:**

This study aims to evaluate the sleep parameters of the Oura ring along with the Samsung Gear Sport watch in comparison with a medically approved actigraphy device in a midterm everyday setting, where users engage in their daily routines.

**Methods:**

We conducted home-based sleep monitoring in which the sleep parameters of 45 healthy individuals (23 women and 22 men) were tracked for 7 days. Total sleep time (TST), sleep efficiency (SE), and wake after sleep onset (WASO) of the ring and watch were assessed using paired *t* tests, Bland-Altman plots, and Pearson correlation. The parameters were also investigated considering the gender of the participants as a dependent variable.

**Results:**

We found significant correlations between the ring’s and actigraphy’s TST (*r*=0.86; *P*<.001*)*, WASO (*r*=0.41; *P*<.001), and SE (*r*=0.47; *P*<.001). Comparing the watch with actigraphy showed a significant correlation in TST (*r*=0.59; *P*<.001). The mean differences in TST, WASO, and SE of the ring and actigraphy were within satisfactory ranges, although there were significant differences between the parameters (*P*<.001); TST and SE mean differences were also within satisfactory ranges for the watch, and the WASO was slightly higher than the range (31.27, SD 35.15). However, the mean differences of the parameters between the watch and actigraphy were considerably higher than those of the ring. The watch also showed a significant difference in TST (*P*<.001) between female and male groups.

**Conclusions:**

In a sample population of healthy adults, the sleep parameters of both the Oura ring and Samsung watch have acceptable mean differences and indicate significant correlations with actigraphy, but the ring outperforms the watch in terms of the nonstaging sleep parameters.

## Introduction

### Background

Sleep is a multifaceted and dynamic phenomenon that indicates individuals’ overall health and well-being and is affected by a variety of factors such as behavioral habits, stress, and disorders [1,2]. Sleep disturbances are common across different population groups (eg, older people and pregnant women) and negatively impact body functions, including the cardiovascular and immune system and hormonal release [3,4]. Such sleep problems need to be investigated thoroughly to reduce the associated health risks and complications. Monitoring sleep quality is a vital step in this regard when the individuals' sleep parameters are tracked [5].

Sleep quality assessment methods have been conventionally performed in clinical settings by monitoring users’ biological signals and body movements. Polysomnography (PSG), the gold standard method used for sleep analysis, is enabled by the continuous monitoring of different cardiorespiratory and neurophysiological indicators [6]. Owing to PSG's complex and multichannel data collection, this method is limited to short-term hospital or laboratory-based monitoring. Actigraphy is another well-established method enabled by a 3D accelerometer that captures the movements of a limb to monitor sleep [7]. This method has been shown to be accurate enough compared with PSG in a healthy subject population [8-11], although the results might be inaccurate when the subjects are individuals with sleep disorders [7,11,12]. In addition, other studies conducted with large populations have shown an agreement between actigraphy and PSG in total sleep time (TST), wake after sleep onset (WASO), and sleep efficiency (SE) parameters [11,13]. On the other hand, some studies have considered the validity of actigraphy’s sleep onset latency (SOL) compared with PSG [11,14] and showed that actigraphy consistently underestimated SOL in comparison with PSG. This method is more convenient than PSG because it allows users to wear the actigraphy device in everyday settings (ie, days to weeks), although conventional medical-grade actigraphy devices are still infeasible for long-term studies (ie, months to years) because of their size, design, and battery life issues.

Advancements in consumer wearable technology provide opportunities to extend sleep monitoring to mid- or long-term home-based health care applications using low-power, miniaturized, and fashionable wearables [15-17]. Wearable electronics and the Internet of Things–based systems are growing dramatically and are expected to revolutionize health care delivery and outcomes [18,19]. In particular, smart rings will most likely become popular in sleep studies. Longer battery life, elegant design, and sophisticated embedded sensors in such rings have enabled them to be used not only in clinical trials (instead of medical-grade actigraphy) but also in different population-based studies [20,21]. Such devices offer continuous data collection of body movements and vital signs in everyday settings. The data can be utilized to continuously monitor sleep disturbances of individuals for an extended period [22].

Sleep monitoring using consumer wearables such as wrist-worn activity trackers, smartwatches, and smart rings necessitate valid sleep data collection and data analysis to provide accurate sleep parameters. Various studies have investigated wrist bands in terms of sleep monitoring accuracy across different population groups. For example, the validation of sleep data of 7 different commercial activity trackers was assessed by conducting data collection for 2 days on healthy adults [23]. In other studies, the sleep estimation of Fitbit devices [24-26], Jawbone [27-29], and physical activity monitors [30] has been investigated against actigraphy, PSG, or both in overnight tests on healthy adolescents and individuals with obstructive sleep apnea. These studies focused on the sleep quality assessment of wearables by tracking a set of nonstaging sleep parameters, including TST, SOL, WASO, and SE [31-34]. Regarding smart ring validation, there is one study that has validated the Oura smart ring against PSG in an overnight laboratory setup [35]; however, there is no previous research in the literature validating a smart ring against actigraphy in the mid- or long-term. Furthermore, these earlier validation studies are limited to laboratory settings and/or overnight (ie, single night) data collection. The effect of home-based health monitoring, where the users might be involved in different conditions and environments, is ignored in these validation studies. Therefore, the results obtained could be inaccurate for long-term and remote monitoring.

### Objectives

In this paper, we aim to assess the validity of sleep data acquired by a smart ring, Oura, in comparison with a medically approved actigraphy device. We utilize the Oura ring as a compact and relatively small device with a user-friendly design. In addition, we assessed the Samsung Gear Sport smartwatch against actigraphy to compare the accuracy of Oura ring in the detection of different sleep attributes. In general, because watches and rings are worn in different parts of the subject’s hand, they respond differently to signal logging disturbances*,* such as *motion artifacts.* The devices were tested in a 7-day monitoring study, approved by the ethical committee, where the sleep data of 45 healthy individuals were monitored. Participants were asked to use the devices 24 hours for 7 days and carry out their daily routines as usual. We compared TST, SOL, WASO, and SE obtained from the Oura ring, Samsung watch, and ActiGraph. The parameters obtained by the 2 consumer-grade wearables (ie, the ring and the watch) were evaluated with the sleep parameters extracted from actigraphy using paired *t* tests, Bland-Altman [36] plots, and Pearson correlation. The parameters were investigated considering the gender of the participants as a dependent variable. Finally, we conclude the paper with a discussion of our obtained results and the validity of sleep data of the wearables in everyday settings.

## Methods

### Participants and Recruitment

Recruitment was performed in southern Finland from July to August 2019. In earlier validation studies of commercial devices, the sample sizes varied between 20 and 40. Therefore, we aimed at a target sample of 40 people. The recruitment started with convenience sampling by personally contacting a few students and staff members of the University of Turku. Afterward, snowball sampling was used until the target sample size was reached; 6 additional participants were enrolled because of expected missing data. We aimed for variation among participants by age, weight, physical activity, education, and lifestyle as related to sleep and stress levels.

A sample of healthy individuals between 18 and 55 years of age was enrolled. Potential participants were excluded if they had (1) a diagnosed cardiovascular disease, (2) restrictions regarding physical activity, (3) symptoms of an illness at the time of recruitment (ie, flu symptoms including sore throat, runny nose, cough, and fever), or (4) any restrictions on using the devices at work. In a face-to-face meeting with the interested individuals, researchers described the purpose of the study and the wearable devices. They were asked to wear the Gear Sport smartwatch, Oura ring, and ActiGraph wristband for 1 week in their normal daily life. Each participant provided written informed consent. Altogether, 46 participants, including 23 women and 23 men, participated in the study. A participant (male) was excluded from the analysis because he did not wear the actigraphy device. Therefore, the final sample size was 45 (23 women, 22 men). Table 1 shows the participants’ background information. The table includes 42 participants, as the background information of the 3 participants is missing.

**Table 1:** Participants’ background information.

Characteristics		Values
**Age (years), mean (SD)**		
	Women	31.5 (6.6)
	Men	33 (6)
**BMI, mean (SD)**		
	Women	24.4 (5.6)
	Men	25.5 (2.9)
**Expected sleep (daily hours), mean (SD)**		
	Women	7.35 (1.00)
	Men	7.17 (1.05)
**Physical activity, n (%)**		
	Almost daily	12 (27)
	Once a week	9 (20)
	>Once a week	21 (47)
**Working status, n (%)**		
	Working	32 (71)
	Unemployed	1 (2)
	Student	8 (18)
	Other	1 (2)

### Ethics

The study was conducted according to the ethical principles based on the Declaration of Helsinki and the Finnish Medical Research Act (#488/1999). The study protocol received a favorable statement from the ethics committee (University of Turku, Ethics committee for Human Sciences, Statement #44/2019). The participants were informed about the study, both orally and in writing, before obtaining their consent. Participation was voluntary, and all participants had the right to withdraw from the study at any time and without giving any reason. To compensate for the time used for the study, each participant received a €20 (US $23) gift card to the grocery store at the end of the monitoring period when returning the devices.

### Data Collection

Our data collection for 1 week included 4 approaches for monitoring participants’ sleep. We utilized 3 devices (ie, 2 wearable and 1 actigraphy device) to continuously capture sleep data and a self-report form by which subjective measures were collected. Samsung Gear and ActiGraph were worn in the wrist, and the Oura ring was worn in one of the fingers of the nondominant hand; thus, all 3 devices were on the same hand. The participants completed a short background questionnaire at the meetings. They were also asked to report their sleep times, such as bedtime, waking up time, and naps, during the 7-day study period via a structured self-report (ie, daily log) form. They were also asked to report other events during the study, such as device removal from the wrist or if specific events occurred (eg, visiting a hospital because of a disease). The self-report data were used to interpret the actigraphy data and mitigate possible errors; such a correction was necessary for this study because the actigraphy was selected as the baseline sleep monitoring method. In addition to the verbal instructions, participants were given a written guideline for using the devices.

The Oura ring [37] was the first wearable device investigated in this study. The Oura ring is a commercial sleep tracker device that uses acceleration and gyroscope data, photoplethysmogram (PPG) signal, and body temperature to estimate sleep parameters, heart rate variability, respiratory rate, and intensity of physical activity. The ring is lightweight (4-6 g) and easy to use. It also has an acceptable battery life, that is, the battery lasts up to 1 week in regular use. The ring is connected to the Android or iOS Oura mobile app via Bluetooth. The data are automatically sent to the mobile app and transferred to the cloud server. The data can be accessed from the mobile app or the cloud server. In this study, we extracted the sleep data of participants from the Oura cloud.

In addition to the Oura ring, we used the Samsung Gear Sport watch [38], which is an open-source smartwatch that enables remote health monitoring. The watch includes a PPG sensor and an inertial measurement unit through which PPG signal, acceleration, and gyroscope data can be collected continuously. The data are processed to extract various variables, including heart rate, sleep duration, and step counts. The Gear Sport watch runs open-source Tizen operating system, enabling customized data collection. In this study, we programmed the watch to collect sleep parameters, PPG data, and hand movement data during the monitoring. The PPG and hand movement data were utilized to validate the sleep events (detailed in the *Data Analysis* section). Moreover, we also developed an app for the watch to send the collected data to our server via Wi-Fi.

For actigraphy, we used the wGT3X-BT device by ActiGraph. The wGT3X is a noncommercial triaxial accelerometer that measures the wrist’s acceleration in 3 orthogonal axes at 80 Hz. This device is waterproof, and its battery life is approximately 3 weeks. The device does not provide any feedback to the participants about their activity or sleep. The acceleration data collected by the device were utilized to obtain the estimates of sleep parameters.

### Data Analysis

Data analysis included the sleep parameter extraction from the collected data and the statistical analysis leveraged to evaluate the ring and watch.

#### Actigraphy

Raw data from the actigraphy device were downloaded to a computer and converted into 60-second epochs using the ActiLife software (version 6.13) [39] provided by the manufacturer (ActiGraph). We used the Cole-Kripke algorithm [40] to define each epoch as sleep or wake. This algorithm was selected because it has been validated in the adult population using wrist-worn accelerometers. The ActiGraph algorithm that is available in the ActiLife software was then used to detect the sleep periods and estimate sleep attributes. Using the Troiano wear time validation algorithm [41], the auto sleep period detection algorithm detects nonwear bouts, ignores nonwear periods greater than a day, and nonwear periods that have almost all zeros (5 or more epochs of nonzeros). The nonwear periods that remain are defined as sleep time. Sleep data were systematically checked, cleaned, and sleep periods that did not represent true sleep times were deleted. These deletions included sleep periods with nonwear time during evenings or mornings that the algorithm had incorrectly scored as sleep, daytime sleep periods, and sleep periods outside the actual measurement week.

#### Wearables

We used the application programming interface provided by the Oura ring and the Samsung watch to extract different semistructured data for our analyses. The Oura ring provides JavaScript Object Notation files, including the sleep parameters per night. The 3 main types of sleep parameters provided by the ring are (1) parameters related to different levels of sleep and nonstaging sleep, including the start and end of sleep, the number of awakenings, total awakening time, and sleep onset, (2) scores to measure the quality of sleep in different stages, and (3) average heart rate for every 5 min during sleep. In this study, we only investigated the nonstaging sleep parameters because of the limitation of the baseline actigraphy method.

In contrast, the Gear Sport watch provides a data record when the user's status changes; for example, the status changes from wake to sleep. We used these records to extract sleep events per night and validated the sleep events using the heart rate and hand movement data collected by the watch. Validation was performed to prevent the misdetection of sleep events owing to not wearing the device. For example, the watch was not used (no movement) for 1 hour, but a sleep event was detected by mistake. In this regard, we recorded a window of 30-second PPG signal when a sleep event started and ended. The sleep event was considered valid if valid heart rate values were detected from the PPG signals. In addition, we considered the hand movement magnitude for validation if the PPG signal was invalid because of practical issues. Finally, we cross-checked the sleep events with the step count data (reported by the watch) and corrected or discarded the sleep events if there was no match between the data.

It should be noted that the watch could not detect a few sleep events because of technical and practical issues during the monitoring. For example, the sleep event was missed because the watch’s turn-off button was pressed accidentally during the night. This issue mostly occurred during the monitoring, as the watch and actigraphy were worn on the same hand close to each other. As the watch could not record the sleep events, we removed 21 nights of data out of 181 (21/181, 11.6%) of the watch for the sake of an unbiased comparison between the actigraphy and watch.

**Figure 1:**
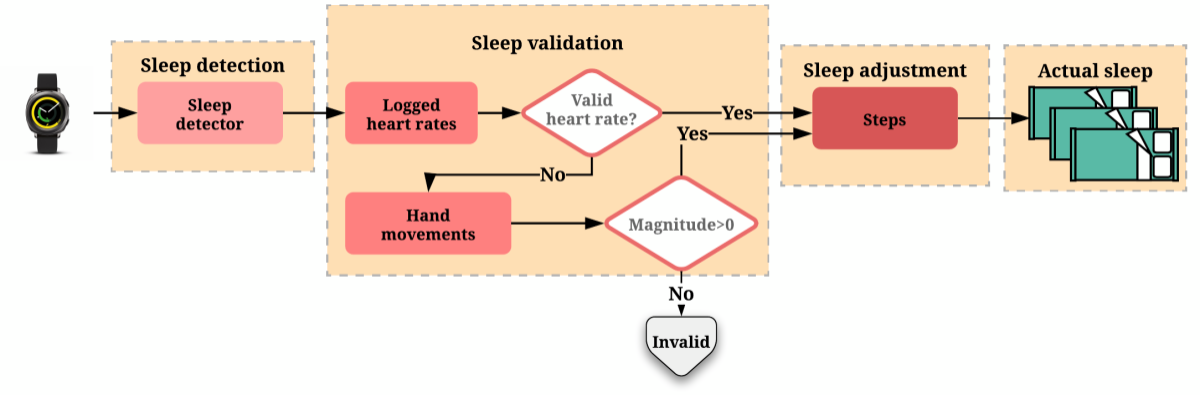
Watch data processing pipeline.

Using the actual valid sleep events, we calculated WASO, TST (in minutes), and SE (%) per night. As the watch does not provide SOL explicitly, we calculate such a feature based on the difference between the start of the actual sleep and the last time the subject had steps. A summary of the processing pipeline is illustrated in Figure 1.

### Statistical Analysis

We report the mean, SD, and 95% CI of the sleep parameters collected by the Oura ring, Samsung watch*,* and ActiGraph*.* The difference between the ring (or the watch) and the ActiGraph was also computed using two-tailed paired *t* tests to test the null hypothesis. In our context, the null hypothesis is that the true mean difference between the two measurements is 0 [42]. Due to the interest in observing the paired differences between values reported by ring (or the watch) and ActiGraph (baseline), the paired *t* test was utilized. In addition, we used the Bland-Altman plot to illustrate and estimate the agreement between the devices. These methods provided mean differences (bias) and SD of the differences between the ring (or the watch) and the actigraphy, lower and upper agreement limits, and 95% CI of the mean differences. The sign of mean differences indicates underestimation or overestimation of the ring (or the watch) compared with the actigraphy: a negative bias shows an overestimation, whereas a positive bias indicates an underestimation.

The satisfactory difference between the ring (or the watch) and the actigraphy data was selected as ≤30 min for TST and WASO and <5% for SE, similar to other studies in the literature [27,35,43]. We investigated the ratio of the samples within these satisfactory ranges. Moreover, we also investigated gender as a dependent variable in the validity of sleep parameters using *t* tests, considering the mean differences between the ring (or the watch) and the actigraphy.

Finally, to analyze the linear relationship between actigraphy and the ring (or the watch) corresponding sleep measurements, we performed Pearson correlation tests on pairwise sleep attributes of the actigraphy and the ring (or the watch).

## Results

### Study Population

A total of 45 subjects (23 women and 22 men) participated in this study. The subjects were 33.1 years old, on average, with an SD of 6.4 years. In total, we recorded 284 valid available days by actigraphy; however, after matching the corresponding available days of the ring (or the watch), we had fewer valid days for the analysis.

As discussed in the *Methods* section, in this study, we exploited 4 different sleep attributes. Although the results regarding SOL are not conclusive (because SOL of the actigraphy is unreliable [14]), for the sake of comparison, we report such results in addition to the other sleep parameters in this section.

### Comparisons Between Ring and Equivalent Actigraphy Sleep Measures

To validate the Oura ring against actigraphy, we matched the available dates of the ring with the corresponding dates of actigraphy. In total, for all the participants, sleep data of 266 days (ie, 5.91, SD 1.32 days per subject) were included in the analysis.

**Figure 2:**
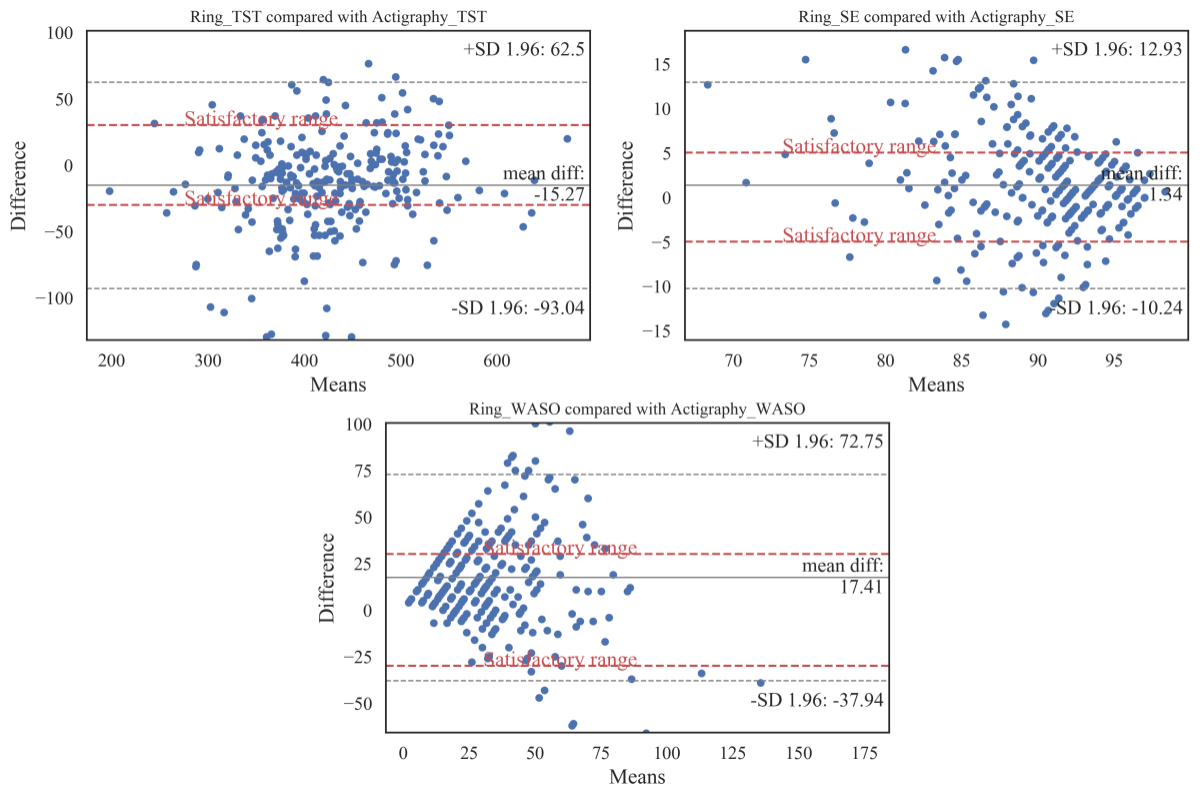
Bland-Altman plots for total sleep time, sleep efﬁciency, and wake after sleep onset gathered by the Oura ring and the actigraphy device. Subjects’ actigraphy minus Oura ring discrepancies on sleep parameters (y-axis) are plotted compared with actigraphy (x-axis). Biases, upper, and lower agreement limits are marked. In addition, the satisfactory ranges are plotted as the dashed lines. SE: sleep efﬁciency; TST: total sleep time; WASO: wake after sleep onset.

The mean, SD, and 95% CI of the extracted sleep parameters are presented in Table 2. The table also shows the paired *t* test values of these parameters with their corresponding *P* values. Bland-Altman plots were used to show the agreements between the 2 measures. Figure 2 depicts the agreement between the ring and actigraphy for the TST, WASO, and SE. The bias and lower and upper agreement limits for these parameters are also summarized in Table 3.

**Table 2:** Mean, SD, 95% CI, and paired t test results for the actigraphy and the Oura ring sleep parameters in a sample of 45 healthy adults.

Parameter		Mean (SD)	95% CI	*t* value (*df*)	*P* value
**Total sleep time (min)**					
	*t* test	N/A^a^	N/A	−6.26 (265)	<.001
	Actigraphy	419.04 (78.31)	409.59-428.5	N/A	N/A
	Oura ring	434.31 (72.14)	425.6-443.02	N/A	N/A
**Sleep efficiency (%)**					
	*t* test	N/A	N/A	3.69 (265)	<.001
	Actigraphy	90.47 (5.1)	89.86-91.09	N/A	N/A
	Oura ring	89.13 (6.28)	88.38-89.89	N/A	N/A
**Wake after sleep onset (min)**					
	*t* test	N/A	N/A	10.03 (265)	<.001
	Actigraphy	43.57 (27.28)	40.28-46.86	N/A	N/A
	Oura Ring	26.17 (24.98)	23.15-29.18	N/A	N/A

^a^N/A: not applicable.

**Table 3:** Bias and agreement limits based on Bland-Altman plots for the actigraphy and the Oura ring.

Parameter	Mean difference (SD)	Lower and upper agreement limits
Total sleep time (min)	−15.27 (39.68)	−93.04, 62.5
Sleep efficiency (%)	1.34 (5.91)	−10.24, 12.93
Wake after sleep onset (min)	17.41 (28.24)	−37.94, 72.75

As shown in Table 2, the ring significantly overestimated the actigraphy (t_265_=−6.26; *P*<.001) in the estimation of TST. On the basis of Figure 2, this overestimation in TST is, on average, 15.27 (SD 39.68) min (95% CI −20.07 to −10.47). Of 266 total samples, 14 fell outside the agreement range (lower limit −93.04 min, upper limit 62.50 min). The mean difference of TST between the actigraphy and ring fell within the satisfactory range, and 65.0% (173/266) of the data samples followed the satisfactory range condition.

On the other hand, in terms of WASO, the Oura ring significantly underestimated (t_265_=10.03; *P*<.001) the actigraphy by, on average, 17.41 min (95% CI 13.99 to 20.82). Out of 266 samples, 17 fell outside the agreement limits (lower limit −37.94 min, upper limit 72.75 min). In terms of the satisfactory range, the mean difference fell within the range and covered 69.9% (186/266) of the total samples.

In addition, the Oura ring underestimated SE compared with the actigraphy by 1.34% on average (95% CI 0.63 to 2.06). This underestimation was significant*,* as shown in Table 2 *(t*_265_=3.69; *P*<.001). The mean difference in SE between the Oura ring and the actigraphy fell within the satisfactory range (<5%), along with 65.8% (175/266) of samples (including 44 out of 45 subjects). Moreover, 18 samples fell outside the agreement limits (lower limit −10.24%, upper limit 12.93%).

### Comparisons Between Watch and Equivalent Actigraphy Sleep Measures

Similar to the ring validation, we considered the available dates for the actigraphy with corresponding data collected by the Samsung watch. As mentioned in the *Wearables* section, we removed the technically invalid watch data that occurred because of practical issues during the monitoring. Therefore, there were fewer sleep data from the watch than the other devices. After the matching procedure and invalid data removal, the number of subjects for the watch validation was 35 (19 men and 16 women), with 134 data samples (3.82, SD 1.50 days per subject). Table 4 summarizes the mean, SD, and 95% CI of the Samsung watch and the actigraphy with the corresponding available dates for different sleep parameters.

**Table 4:** Mean, SD, 95% CI, and paired t test results for the actigraphy and the Samsung watch sleep parameters in a sample of 35 healthy adults.

Parameter		Mean (SD)	95% CI	*t* value (*df*)	*P* value
**Total sleep time (min)**					
	*t* test	N/A^a^	N/A	−3.54 (133)	<.001
	Actigraphy	409.29 (81.43)	395.38-423.21	N/A	N/A
	Samsung watch	431.81 (82.21)	417.76-445.85	N/A	N/A
**Sleep efficiency (%)**					
	*t* test	N/A	N/A	−6.49 (133)	<.001
	Actigraphy	90.40 (5.05)	89.54-91.26	N/A	N/A
	Samsung watch	94.84 (7.03)	93.64-96.04	N/A	N/A
**Wake after sleep onset (min)**					
	*t* test	N/A	N/A	10.26 (133)	<.001
	Actigraphy	42.23 (23.43)	38.23-46.24	N/A	N/A
	Samsung watch	10.96 (30.46)	5.76-16.17	N/A	N/A

^a^N/A: not applicable.

**Figure 3:**
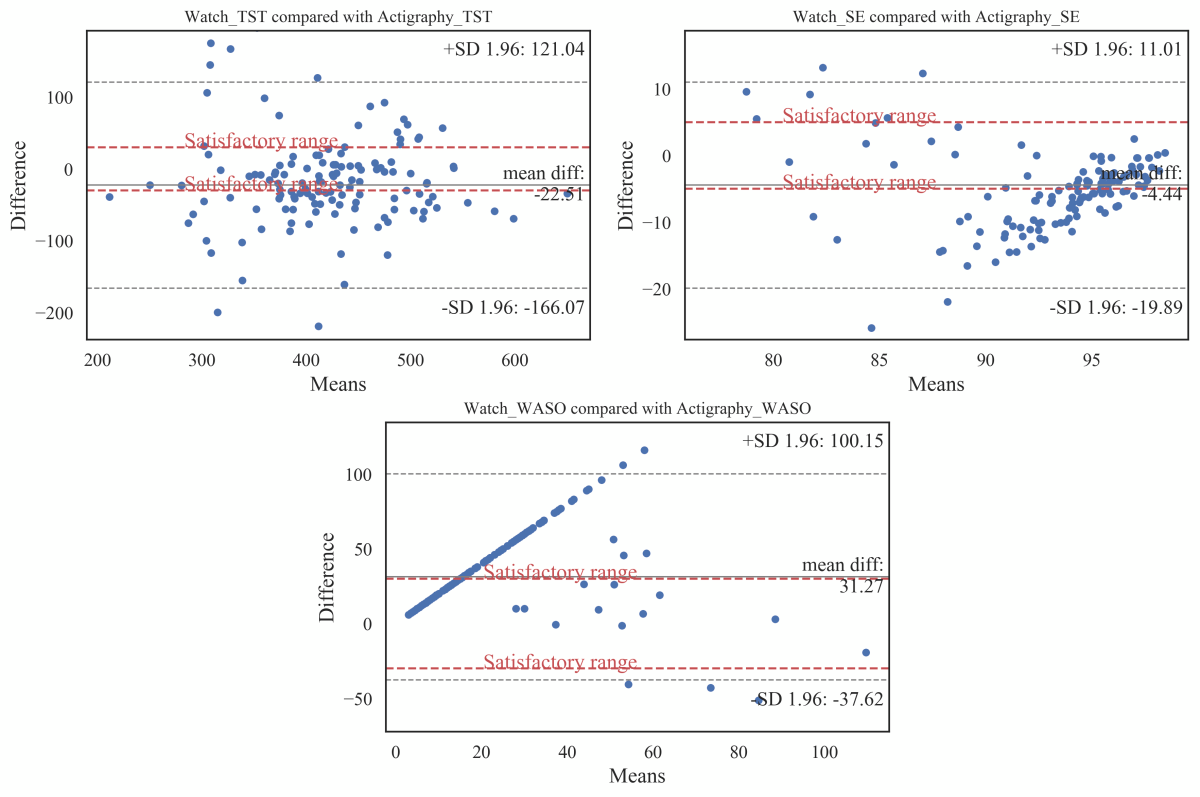
Bland-Altman plots for total sleep time, sleep efﬁciency, and wake after sleep onset gathered by the Samsung watch and the actigraphy device. Subjects’ actigraphy minus Samsung watch discrepancies on sleep parameters (y-axis) are plotted compared with actigraphy (x-axis). Biases, upper, and lower agreement limits are marked. In addition, the satisfactory ranges are plotted as the dashed lines. SE: sleep efﬁciency; TST: total sleep time; WASO: wake after sleep onset.

In addition, we performed paired *t* tests for the sleep parameters of the 2 devices. The results are shown in Table 4. As shown in this table, the *t* test values for all considered sleep parameters were statistically significant (*P*<.001). The positive and negative sign of the *t* value denotes the underestimation and overestimation of actigraphy by the watch, respectively. Bland-Altman plots showing TST, WASO, and SE agreements between the actigraphy and the watch are also illustrated in Figure 3. Moreover, bias and lower and upper agreement limits of sleep parameter outcomes by the actigraphy and the watch are summarized in Table 5.

**Table 5:** Bias and agreement limits based on Bland-Altman plots for the actigraphy and the Samsung watch.

Parameter	Mean difference (SD)	Lower and upper agreement limits
Total sleep time (min)	−22.51 (73.24)	−166.07, 121.04
Sleep efficiency (%)	−4.44 (7.88)	−19.89, 11.01
Wake after sleep onset (min)	31.27 (35.15)	−37.62, 100.15

As shown in Figure 3, the watch overestimated the actigraphy in TST, on average, by 22.51 min (95% CI −35.08 to −9.95). Among the 134 samples, 9 were beyond the agreement limits (lower limit −166.07 min, upper limit 121.04 min). The mean difference of the actigraphy’s and the watch’s TST was within the satisfactory range; however, less than 50% (52/134, 38.8%) of the samples were within this satisfactory range.

In addition to TST, the Samsung watch overestimated SE by 4.44% (95% CI −5.79 to −3.09) compared with the actigraphy; 8 samples fell outside the agreement limits (lower limit −19.89%, upper limit 11.01%), with 42.5% (57/134) of the samples within the satisfactory range.

On the other hand, the watch underestimated WASO by 31.27 min on average (95% CI 25.24 to 37.3). Only 9 samples were outside of the agreement limits (lower limit −37.62 min, upper limit 100.15 min), and 45.5% (61/134) of the samples were within the satisfactory range.

### Gender-Dependent Changes in the Mean Differences Between the Actigraphy and the Ring (or the Watch)

We also considered the gender of the participants to determine if the mean difference in sleep parameters differed between female and male groups. Table 6 shows the mean and SD of each sleep attribute of the actigraphy and the ring and the difference between these devices for male and female groups, separately.

The average of the mean difference between the TST of the actigraphy and the Oura ring did not differ between the male and female groups (t_530_=0.99; *P*=.32). However, the mean differences of the other sleep parameters (ie, SE and WASO) were significant between female and male participants (*P*<.001 and *P*=.004).

**Table 6:** Mean, SD, and average mean differences (the actigraphy minus the Oura ring) for 23 women (141 samples) and 22 men (125 samples).

Parameter		Mean (SD)			*t* value (*df*)	*P* value
		Actigraphy	Oura ring	Differences		
**Total sleep time (min)**						
	*t* test	N/A^a^	N/A	N/A	0.99 (530)	.32
	Women	429.67 (70.25)	442.66 (64.67)	−12.98 (37.94)	N/A	N/A
	Men	407.05 (85.21)	424.89 (78.94)	−17.84 (41.39)	N/A	N/A
**Sleep efficiency (%)**						
	*t* test	N/A	N/A	N/A	−4.33 (530)	<.001
	Women	90.64 (4.93)	90.73 (5.16)	−0.09 (5.86)	N/A	N/A
	Men	90.29 (5.31)	87.33 (6.9)	2.96 (5.55)	N/A	N/A
**Wake after sleep onset (min)**						
	*t* test	N/A	N/A	N/A	2.86 (530)	.004
	Women	44.9 (30.08)	22.87 (20.7)	22.03 (29.19)	N/A	N/A
	Men	42.07 (23.75)	29.88 (28.69)	12.19 (26.16)	N/A	N/A

^a^N/A: not applicable.

Similarly, we compared the mean differences of the sleep parameters between the actigraphy and the watch for the male and female groups. Table 7 summarizes such differences for each sleep parameter. As shown in Table 7, there was a significant difference between the mean differences of the male and female groups for TST (*P*<.001), SE (*P*=.01), and WASO (*P*=.01).

**Table 7:** Mean, SD, and average mean differences (the actigraphy minus the Samsung watch) for 16 women (65 samples) and 19 men (69 samples).

Parameter		Mean (SD)			*t* value (*df*)	*P* value
		Actigraphy	Samsung watch	Differences		
**Total sleep time (min)**						
	*t* test	N/A^a^	N/A	N/A	3.48 (266)	<.001
	Women	427.08 (73.76)	427.67 (74.76)	−0.59 (65.67)	N/A	N/A
	Men	392.54 (85.22)	435.7 (89.04)	−43.16 (74.01)	N/A	N/A
**Sleep efficiency (%)**						
	*t* test	N/A	N/A	N/A	2.39 (266)	.01
	Women	90.82 (4.88)	93.6 (7.92)	−2.78 (8.04)	N/A	N/A
	Men	90.0 (5.2)	96.01 (5.9)	−6.0 (7.4)	N/A	N/A
**Wake after sleep onset (min)**						
	*t* test	N/A	N/A	N/A	−2.40 (266)	.01
	Women	42.49 (24.33)	18.64 (39.75)	23.85 (42.54)	N/A	N/A
	Men	41.99 (22.73)	3.73 (14.76)	38.26 (24.36)	N/A	N/A

^a^N/A: not applicable.

### Correlations

We also investigated the possible linear relationship between the actigraphy and the ring (or the watch) data, using the Pearson correlation test. The correlation value (*r*) ranges from −1 to 1, where ±1 implies an exact linear relationship. The correlation values and their *P* values are shown in Table 8.

**Table 8:** Pearson correlation between the actigraphy, ring, and smartwatch with the corresponding *P* values for the considered sleep attributes.

Devices	Pearson correlation with the actigraphy, *r*					
	TST^a^	*P* value	SE^b^	*P* value	WASO^c^	*P* value
Oura ring	0.86	<.001	0.47	<.001	0.41	<.001
Samsung watch	0.59	<.001	0.17	.04	0.16	.06

^a^TST: total sleep time.

^b^SE: sleep efficiency.

^c^WASO: wake after sleep onset.

As shown in Table 8, comparing TST of actigraphy with TST of the ring and TST of the watch, we found a significantly high correlation between the actigraphy and the ring (*r*=0.86; *P*<.001). In contrast, the correlation between the actigraphy and the watch was *r*=0.59 (*P*<.001).

With regard to SE, there was a correlation between actigraphy and the ring (*r*=0.47; *P*<.001). In addition, the correlation between the actigraphy and the watch was acceptable (*r*=0.17; *P*=.04), but not as high as that of the ring.

For the WASO validation, there was a significant correlation between the actigraphy and the ring (*r*=0.41; *P*<.001). However, our analysis showed a nonsignificant correlation between WASO of the actigraphy and WASO of the watch (*r*=0.16; *P*=.06).

### SOL Comparison Across Devices

**Figure 4:**
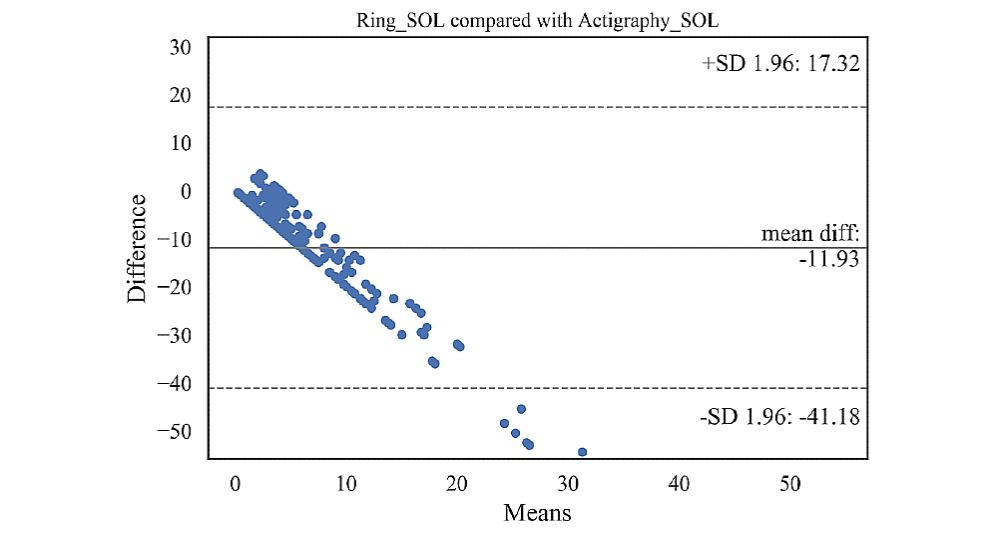
Bland-Altman plot for sleep onset latency estimated by the Oura ring. SOL: sleep onset latency.

**Figure 5:**
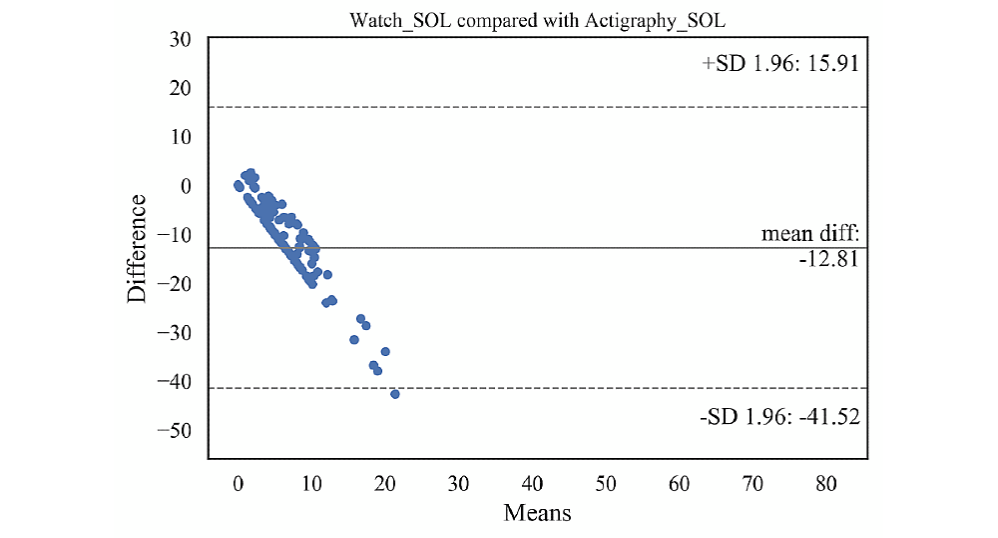
Bland-Altman plot for sleep onset latency estimated by the Samsung watch. SOL: sleep onset latency.

As previously mentioned, SOL results were not conclusive since SOL of actigraphy is unreliable. We report SOL separately in the following: mean, SD, 95% CI, and paired *t* test results of the SOL for comparison between the actigraphy and the Oura ring (or Samsung watch) are presented in Tables 9 and 10. Bland-Altman plots showing the SOL agreements between the actigraphy and the ring (or the watch) are illustrated in Figures 4 and 5. Details of these plots are summarized in Tables 11 and 12.

**Table 9:** Mean, SD, 95% CI, and paired t test results for the actigraphy versus Oura ring estimates of sleep onset latency.

Parameter		Mean (SD)	95% CI	*t* value (*df*)	*P* value
**Sleep onset latency (min)**					
	*t* test	N/A^a^	N/A	−13.01 (265)	<.001
	Actigraphy	0.91 (1.37)	0.75-1.08	N/A	N/A
	Oura ring	12.84 (14.92)	11.04-14.65	N/A	N/A

^a^N/A: not applicable.

**Table 10:** Mean, SD, 95% CI, and paired t test results for the actigraphy versus Samsung watch estimates of sleep onset latency.

Parameter		Mean (SD)	95% CI	*t* value (*df*)	*P* value
**Sleep onset latency (min)**					
	*t* test	N/A^a^	N/A	−10.08 (133)	<.001
	Actigraphy	0.99 (1.38)	0.75-1.22	N/A	N/A
	Samsung watch	13.79 (14.86)	11.25-16.33	N/A	N/A

^a^N/A: not applicable.

**Table 11:** Bias and agreement limits based on Bland-Altman plot of the sleep onset latency for the actigraphy and the Oura ring.

Parameter	Mean difference (SD)	Lower and upper agreement limits
Sleep onset latency (min)	−11.93 (14.92)	−41.18, 17.32

**Table 12:** Bias and agreement limits based on Bland-Altman plot of sleep onset latency for the actigraphy and Samsung watch.

Parameter	Mean difference (SD)	Lower and upper agreement limits
Sleep onset latency (min)	−12.81 (14.65)	−41.52, 15.91

The Oura ring overestimated the SOL, on average, by 11.93 min (95% C: −13.74 to −10.13) compared with the actigraphy. Out of 266 samples, 14 fell outside the agreement limits (lower limit −41.18 min, upper limit 17.32 min). Table 9 shows that the overestimation of the SOL by the ring was significant (t_265_=−13.01; *P*<.001). Similarly, the watch overestimated the SOL, on average, by 12.81 min (95% CI −15.32 to −10.29). Most of the samples (all except 2) were within the agreement limits (lower limit −41.52 min, upper limit 15.91 min).

## Discussion

### Principal Findings

To the best of our knowledge, this is the first sleep validation study of the Oura ring and the Samsung watch performed under free-living conditions in comparison with an actigraphy method. The free-living condition allows participants to engage in their daily routines as usual during the monitoring. If commercial devices are used in trials under such free-living conditions, subjective evaluations and self-reports are insufficient to measure the validity of these devices [44-46]. It is important to test these devices against research devices to investigate their error margins and to standardize their software versions, minimizing controllable measurement differences. In contrast to related work, this study investigated wearables in a 1-week home-based monitoring, providing a higher confidence level on the validity of sleep parameters reported by these wearables. We discuss the results obtained and compare them with the related sleep validation studies, most of which are limited to the laboratory settings and compared with PSG.

Our findings showed that the mean differences of TST, WASO, and SE between the actigraphy device and the Oura ring were within the satisfactory range (ie, ≤30 min for TST and WASO and <5% for SE). Within the 266 valid total nights of sleep, only 14 TST, 17 WASO, and 18 SE fell outside the agreement limits. Our results also indicated significant correlations between the TST, WASO, and SE of the ring and the actigraphy. These findings are in accordance with a previous validation study of the Oura ring carried out in a single laboratory overnight study [35].

On the other hand, we found significant differences between the means of TST, WASO, and SE of the ring and the actigraphy. In our study, the Oura ring overestimated the TST (15.27 min) and underestimated the WASO (17.41 min) and SE (1.34%). Although the differences were within the satisfactory range, our results showed more overestimation and underestimation of the Oura ring than the lab-based sleep validation study [35]. This might be explained by the difference between the studies’ samples and setups. Our study included more sleep data (ie, 225 more nights) and was performed in the house. Therefore, our results should be more accurate and have higher confidence levels in real-world applications. Unfortunately, these inaccuracies in sleep measurements in commercial devices might decrease their feasibility for clinical trials [47].

In accordance, the results showed biases in the sleep parameters provided by the Oura ring. However, the mean differences were within the satisfactory range, and only a few samples were outside the agreement limits. Therefore, the Oura ring can be acceptable for monitoring nonstaging sleep parameters under free-living conditions.

Moreover, our results indicated that the mean differences of the TST, WASO, and SE between the Samsung watch and the actigraphy were higher than the Oura ring’s mean difference. The TST and SE mean differences of the watch were higher but still within the satisfactory range. However, the WASO mean difference (ie, 31.27 min) was negligibly higher than the range. Within the 134 valid total nights of sleep detection by the watch, 9 TST, 9 WASO, and 8 SE fell outside the agreement limit. Similarly, the correlation of the watch and actigraphy was lower than the ring, as the Pearson *r* values of the three parameters were closer to 0. Consequently, the sleep parameters of the watch had acceptable mean differences and indicated significant correlations with the actigraphy, but the Oura ring outperforms the Samsung watch in terms of the nonstaging sleep parameters.

### Comparison With Prior Work

In previous studies, wrist activity trackers such as Fitbit Charge HR and Jawbone UP were compared with the PSG in lab tests on healthy adults [24,27,30]. The devices showed good agreement with the PSG in terms of TST, WASO, and SE. This is in accordance with our results for both the Oura ring and the Samsung watch. However, the overestimations or underestimations in our findings were higher than those in previous studies. The biases are particularly significant for the Samsung watch. For example, de Zambotti et al [24] indicated that the Fitbit Charge HR overestimates TST by 8 min and SE by 1.8% and underestimates WASO by 5.6 min. These low biases might be because of their limited setups and data collection, that is, an overnight laboratory sleep test on 32 healthy individuals.

There are a few studies performed under free-living conditions to evaluate activity trackers such as the Misfit Shine, Jawbone UP, and different models of Fitbit on healthy adults [23,48]. Our results regarding the Oura ring highlighted the high correlations obtained by these studies. For instance, Liang et al [48] indicated that there were high Pearson correlations between Fitbit Charge 2 and their baseline (a single-channel electroencephalogram-based device) in terms of TST (*r*=0.94), WASO (*r*=0.25), and SE (*r*=0.50). Ferguson et al [23] considered the TST correlations between four activity tracker devices and a research-grade accelerometer or multi-sensor device (BodyMedia SenseWear). The authors showed that the correlations were higher than 0.82 for the devices. On the other hand, our smartwatch results showed moderate correlations for TST, WASO, and SE.

Furthermore, we considered gender as a dependent variable to evaluate whether there was a mean difference in sleep parameter changes between male and female groups. Considering the Oura ring, our results showed a nonsignificant difference between female and male groups in TST, which is similar to the findings of de Zambotti et al [27]. Moreover, Carter et al [49] evaluated the objective estimation of sleep parameters compared with subjective assessments. In comparison with this study, we obtained similar results in terms of objective TST. However, the watch in our study showed a significant difference in TST. Besides, both the ring and the watch indicated significant differences between female and male groups in WASO and SE, which disagrees with de Zambotti et al [27] but confirms the findings of Carter et al [49].

### Limitations

We considered using an actigraphy device as the baseline method, which is one of the limitations of this study. Our analysis was limited to TST, WASO, and SE parameters. Although we collected the SOL of the Oura ring and the Samsung watch, we could not evaluate the values, as the SOL measure of the actigraphy is unreliable [14]. The actigraphy methods are insufficient for evaluation of sleep stages (eg, deep sleep). Therefore, future work should investigate the sleep stages provided by the ring and watch, considering a feasible PSG or electroencephalogram-based method designed for home-based monitoring.

Another limitation of this study is that only healthy participants were included in the analysis. However, other studies have shown that the accuracy of the wearables might differ for different population groups [29,34]. This issue may limit the generalizability of the findings. This study’s future directions are to perform a home-based sleep validation study to assess the accuracy of wearables for population groups of different ages (eg, adolescents and older people) and sleep disorders (eg, obstructive sleep apnea). Besides, bed-based and ballistocardiograph-based sensors [50] can be used to mitigate user errors during data collection.

### Conclusions

Sleep monitoring in free-living conditions becomes feasible and practicable using commercial devices such as smart rings and smartwatches. Notwithstanding the advances and feasibility of these wearables, their validity in terms of sleep parameters was not thoroughly investigated, especially for mid- to long-term studies in everyday settings. This study assessed the Oura ring and the Samsung Gear Sport watch by examining their TST, WASO, and SE under free-living conditions. The wearable devices were tested in home-based monitoring, where the sleep parameters of 45 healthy participants were tracked for 7 days. The assessment was performed in comparison with an actigraphy device, leveraging the paired *t* tests, Bland-Altman plots, and Pearson correlations. Sleep parameters were investigated considering the gender of the participants as a dependent variable. Our results showed that despite the statistically significant differences in the sleep parameters (ie, TST, WASO, and SE) of both the Oura ring and the Samsung watch compared with the actigraphy device, the mean differences were within the satisfactory ranges. The sleep parameters also indicated significant correlations with actigraphy. Besides, we showed that there was no significant difference in the validation of TST between male and female groups in the Oura ring; however, both the Oura ring and the Samsung watch indicated significant differences between the female and male groups in the estimation of WASO and SE.

Similarly, in a population sample of healthy adults, both the Oura ring and the Samsung watch had acceptable mean differences and indicated significant correlations with the actigraphy. However, the biases of the ring were considerably lower than the biases of the watch. Further validation is required to assess the validity of the sleep stages provided by the ring and the watch under free-living conditions. Moreover, future work should include the assessment of the devices for other population groups, such as individuals with sleep disorders.

## Abbreviations

PPG: photoplethysmogram
PSG: polysomnography
SE: sleep efficiency
SOL: sleep onset latency
TST: total sleep time
WASO: wake after sleep onset
